# Supplementary material for: How Realistic Are Idealized Copper Surfaces? A Machine Learning Study of Rough Copper–Water Interfaces
Source: ACS Mater Au. 2026 Jan 13;6(2):379–89. doi: 10.1021/acsmaterialsau.5c00174 (PMC12983105; doi:10.1021/acsmaterialsau.5c00174)
Supplement: Supplementary file 1 [file mg5c00174_si_001.pdf]

Supporting Information: How Realistic are Idealized Copper Surfaces? A Machine Learning Study of Rough Copper-Water Interfaces

Linus C. Erhard,[*,†,‡] Johannes Schörghuber,[†,‡] Aleix Comas-Vives,[†] and Georg K. H. Madsen[*,†]

†Institute of Materials Chemistry, TU Wien, A-1060 Vienna, Austria

‡These authors contributed equally

E-mail: linus.erhard@tuwien.ac.at; georg.madsen@tuwien.ac.at

Table S1: **Detailed simulation protocol to produce rough copper surfaces by nanoparticles placed on top.**We used two different temperature protocols for producing rough surfaces. In version 1, we used lower temperatures of maximum 1100 K, but therefore longer simulation times. In version 2, we used higher temperatures of a maximum of 1300 K, but for much shorter times. The temperature of the bottom part of the slab was always fixed at 500 K. In an intermediated region of 10 Å we used starting from the second step a NVE ensemble to allow for a temperature gradient.

Protocol	Simulation Time [ps]	Temperature [K] Top Layer	Temperature [K] Bottom Layer	Box Change Allowed in Surface Plane
Version 1				
Step 1.	20	500	500	Yes
Step 2.	200	500→ 1100	500	No
Step 3.	400	1100	500	No
Step 4.	4000	1100 → 500	500	No
Version 2				
Step 1.	20	500	500	Yes
Step 2.	20	500→ 1300	500	No
Step 3.	10	1300	500	No
Step 4.	20	1300 → 900	500	No
Step 5.	1000	900 → 500	500	No

Table S2: **Detailed simulation protocol to produce rough copper surfaces by inserting indenters.** The whole simulation is performed at constant volume conditions. We start by equilibrating the system and then increasing the temperature of the top layer. Between the top layer and the bottom layer is a 10 Å thick region, where we do not apply a thermostat. The roughness of the surface is then induced by indenters inserted from above the slab into the top layer. The indenters are implemented by dummy particles, which interact by a purely repulsive Lennard-Jones potential with the copper. The radius of the indenters, as well as the intrusion depth, is varied randomly for different structures. We apply a reflecting wall at the bottom and well above the top of the slab to prevent atoms from escaping. After inserting, we keep the indenters at a constant position in the simulation, while we cool down the top layer.

Protocol	Simulation Time [ps]	Temperature [K] Top Layer	Temperature [K] Bottom Layer	Indenters Active	Indenters Moving Down
Step 1.	10	500	500	No	No
Step 2.	40	500→ 1500	500	No	No
Step 3.	200	1500	500	Yes	Yes
Step 4.	40	1500	500	Yes	No
Step 5.	2000	1500 → 500	500	Yes	No

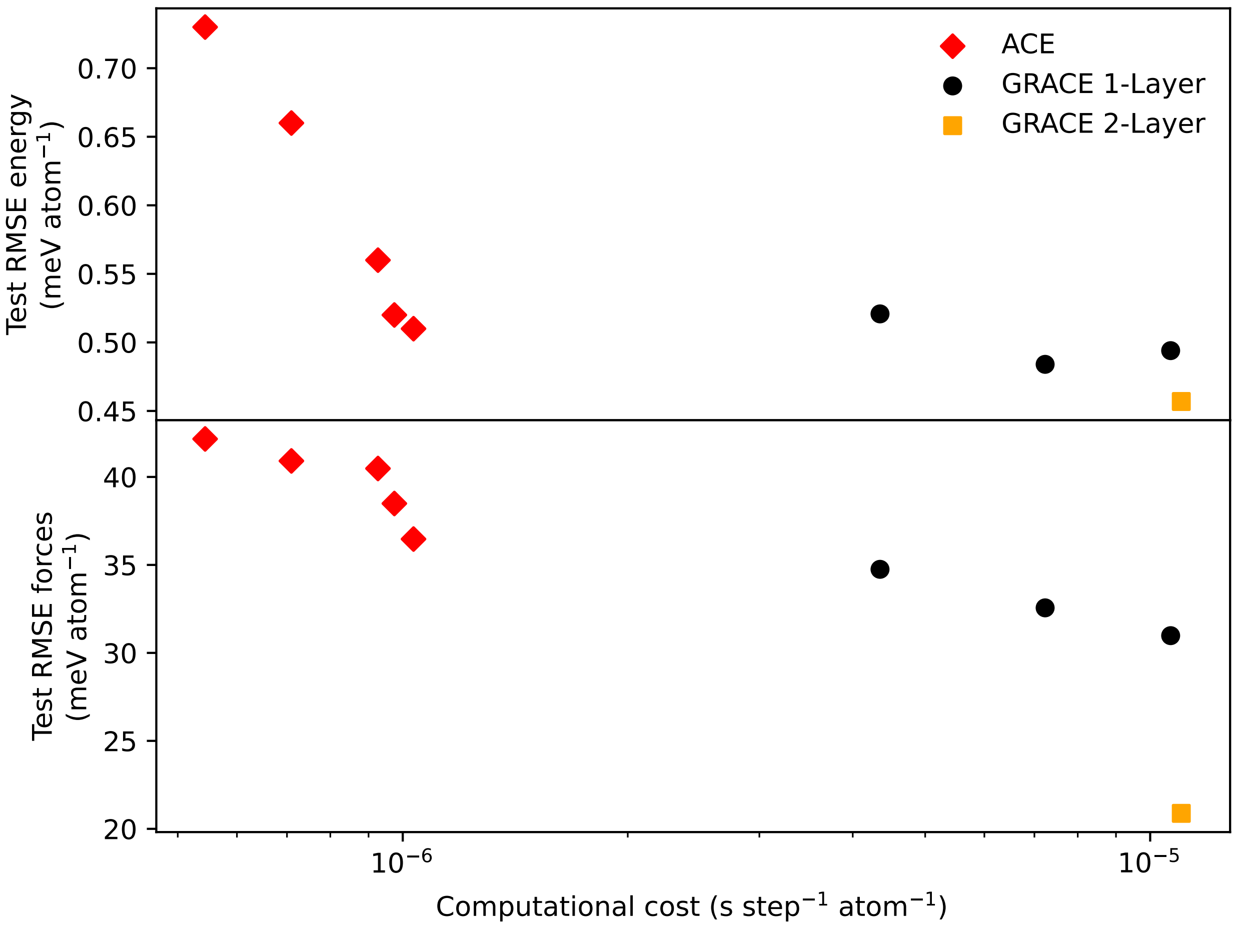

Figure S1: **Accuracy and computational costs of ACE and different types of GRACE.** ACE and GRACE 1-Layer are compared with regard to their accuracy and their computational efficiency to GRACE 2-Layer. In the case of ACE, we varied the cutoff radius and the number of basis functions, in the case of GRACE 1-Layer, we varied only the cutoff radius, leading to several points in the plot. The test set used for the evaluations was sampled during the active learning iterations for rough surfaces by selecting configurations slightly below the uncertainty threshold, yielding 38 configurations in total. This set was then combined with the test set constructed in Ref. 14.

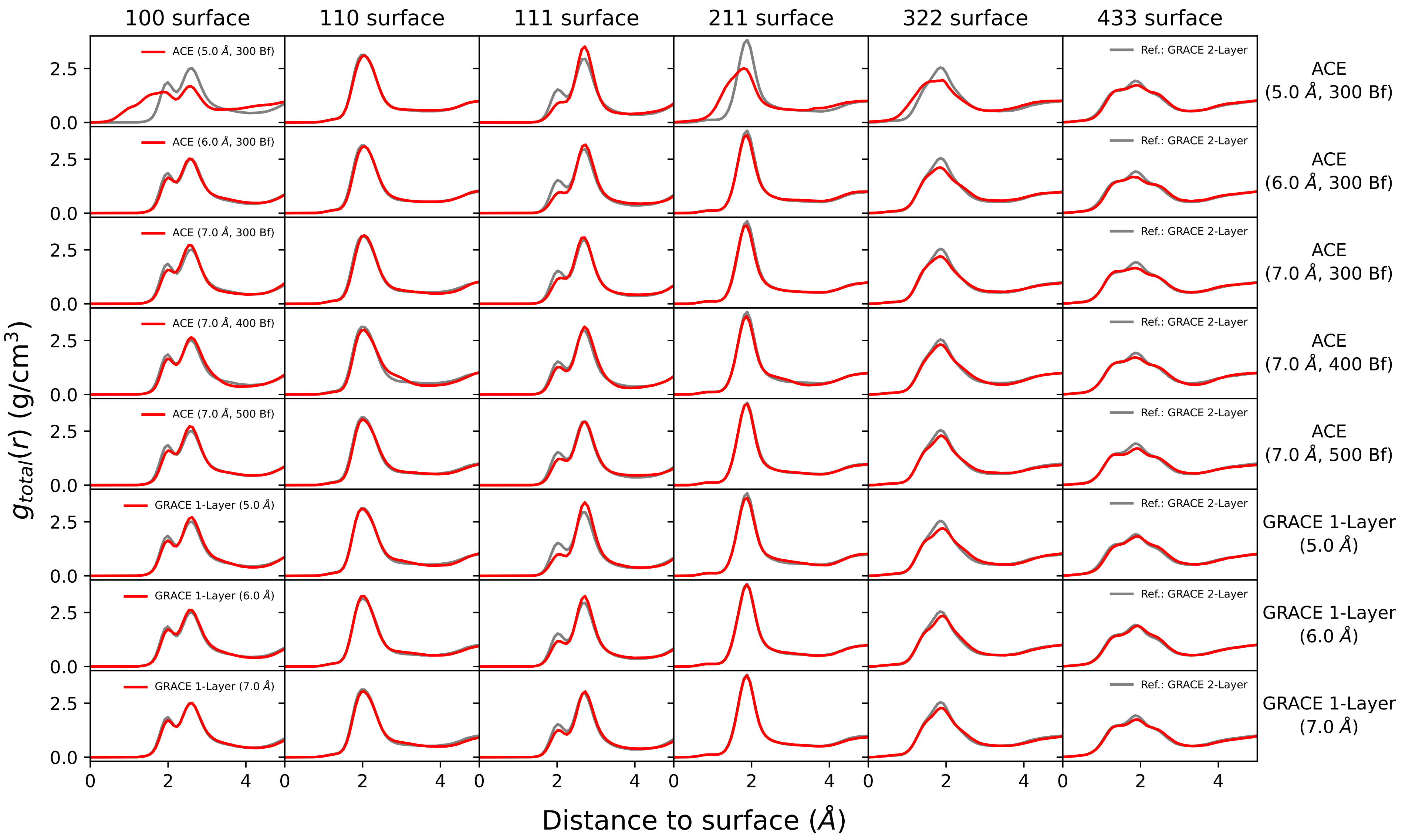

Figure S2: **Density of water as a function of the distance to various copper surfaces.** These plots show how water is distributed as a function of the distances on the copper 100, 110, 111, 211, 322, and 433 surfaces. We evaluated the curves with various machine-learning interatomic potentials, e.g., ACE, GRACE 1-Layer, and GRACE 2-Layer. All potentials have been fitted to the same databases. As GRACE 2-Layer provides the highest accuracy (see Figure S1), we used it in all plots as a reference line (gray).

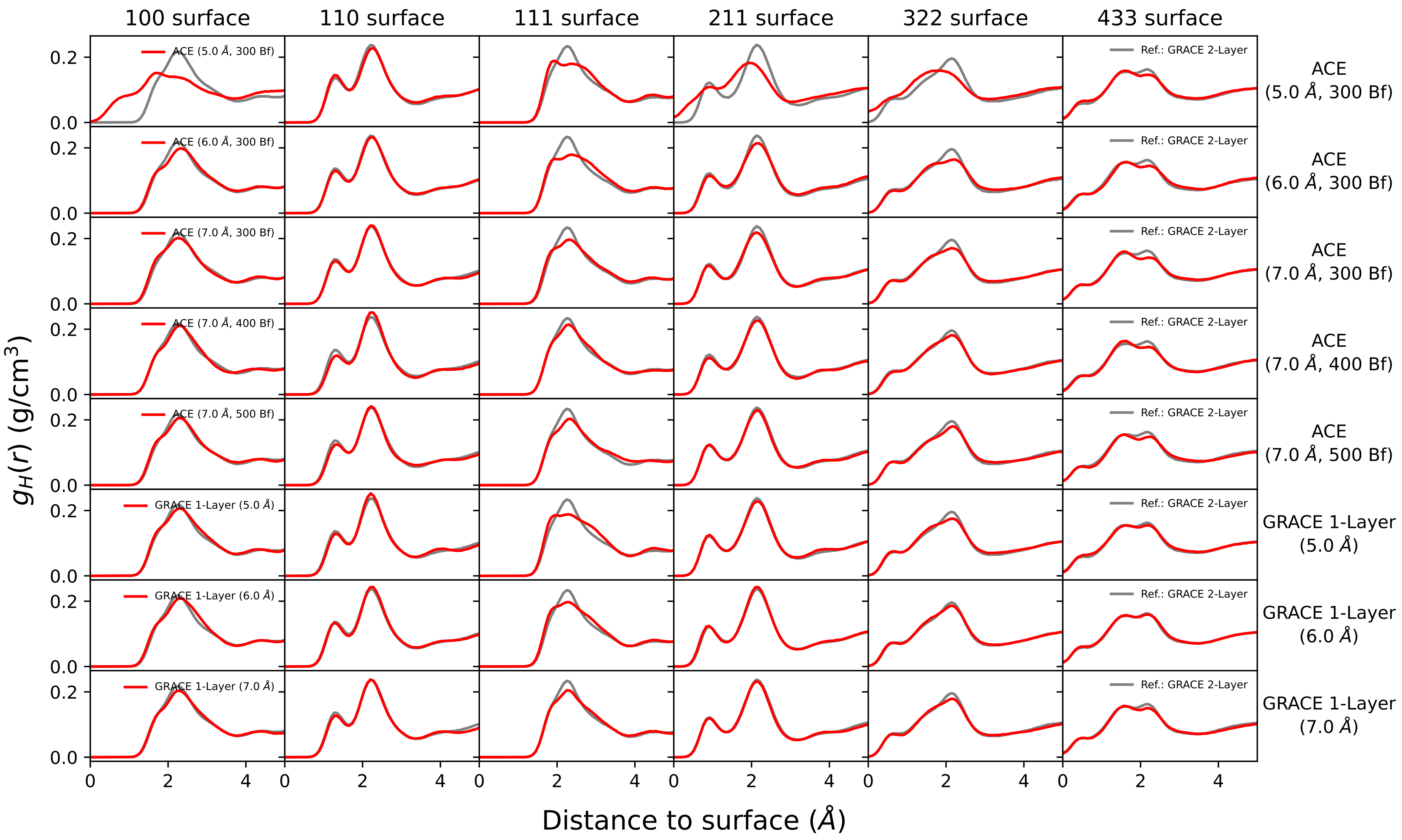

Figure S3: **Density of hydrogen in water as a function of the distance to various copper surfaces.** These plots show how hydrogen, as part of the water molecules, is distributed as a function of the distances on the copper 100, 110, 111, 211, 322, and 433 surfaces. We evaluated the curves with various machine-learning interatomic potentials, e.g., ACE, GRACE 1-Layer, and GRACE 2-Layer. All potentials have been fitted to the same databases. As GRACE 2-Layer provides the highest accuracy (see Figure S1), we used it in all plots as a reference line (gray).

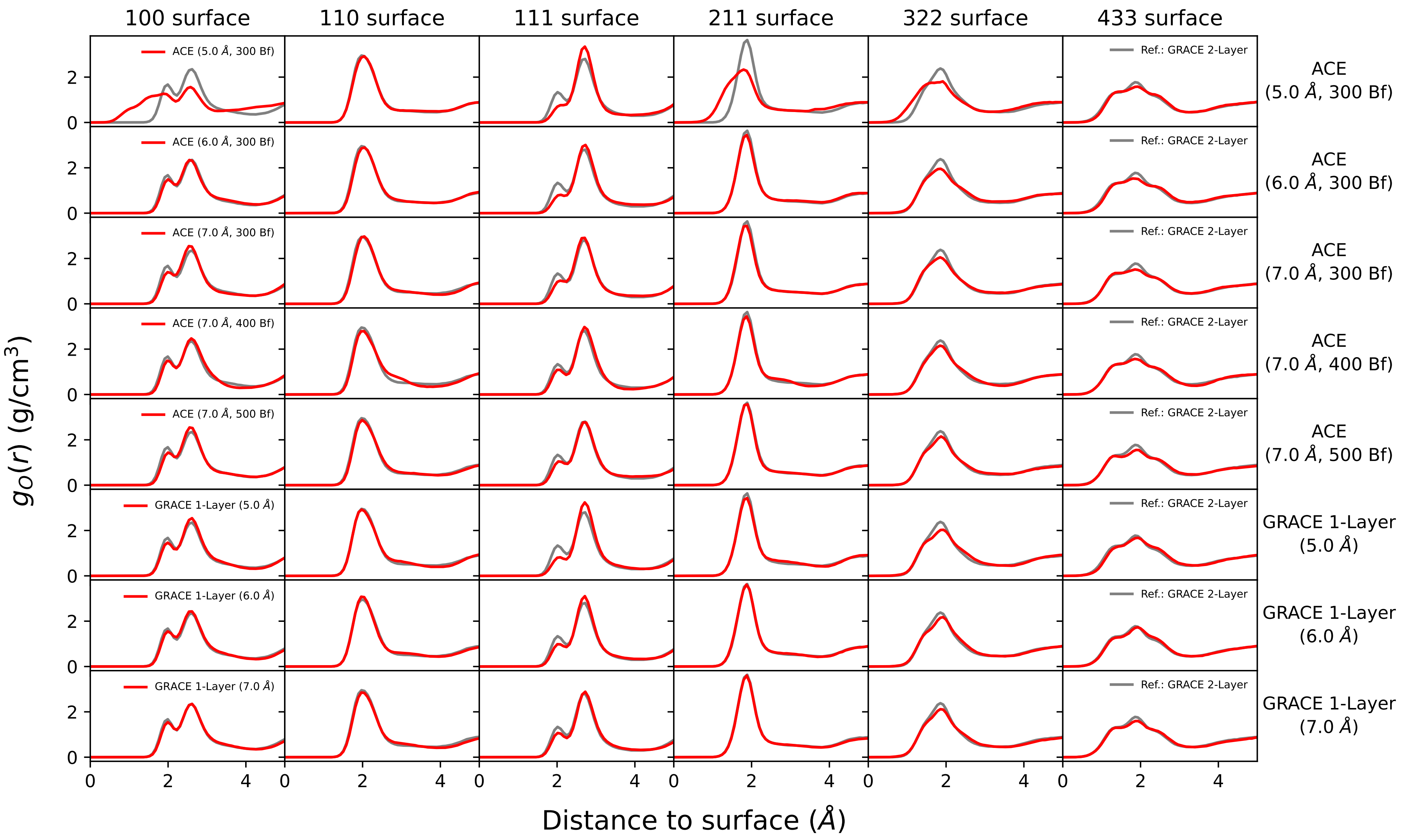

Figure S4: **Density of oxygen in water as a function of the distance to various copper surfaces.** These plots show how oxygen, as part of water molecules, is distributed as a function of the distances on the copper 100, 110, 111, 211, 322, and 433 surfaces. We evaluated the curves with various machine-learning interatomic potentials, e.g., ACE, GRACE 1-Layer, and GRACE 2-Layer. All potentials have been fitted to the same databases. As GRACE 2-Layer provides the highest accuracy (see Figure S1), we used it in all plots as a reference line (gray).

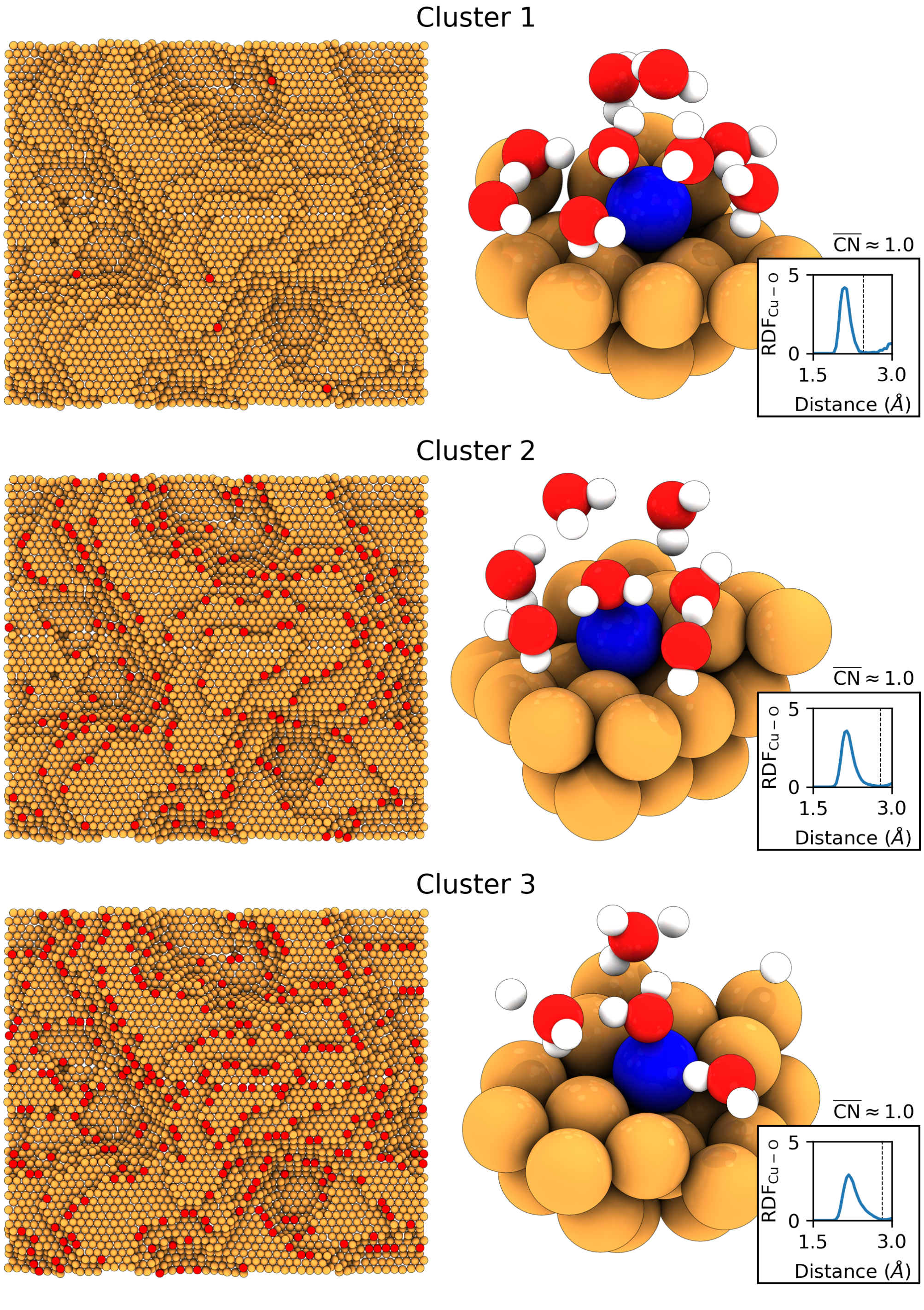

Figure S5: **Rough copper surface with atoms marked according to their cluster classification.** The left side of the figure depicts the rough copper surface, with atoms colored according to their cluster assignment as specified in Figure 6. On the right, an exemplary environment within a 5 Å radius of a cluster atom (marked in blue) is shown. In this representation, copper atoms are colored brown, oxygen atoms are colored red, and hydrogen atoms are colored white. Additionally, the average radial distribution function between copper atoms in this cluster and oxygen atoms is plotted. The average coordination number of copper with respect to oxygen is determined by integrating the first peak of the radial distribution function up to the point indicated by the black line.

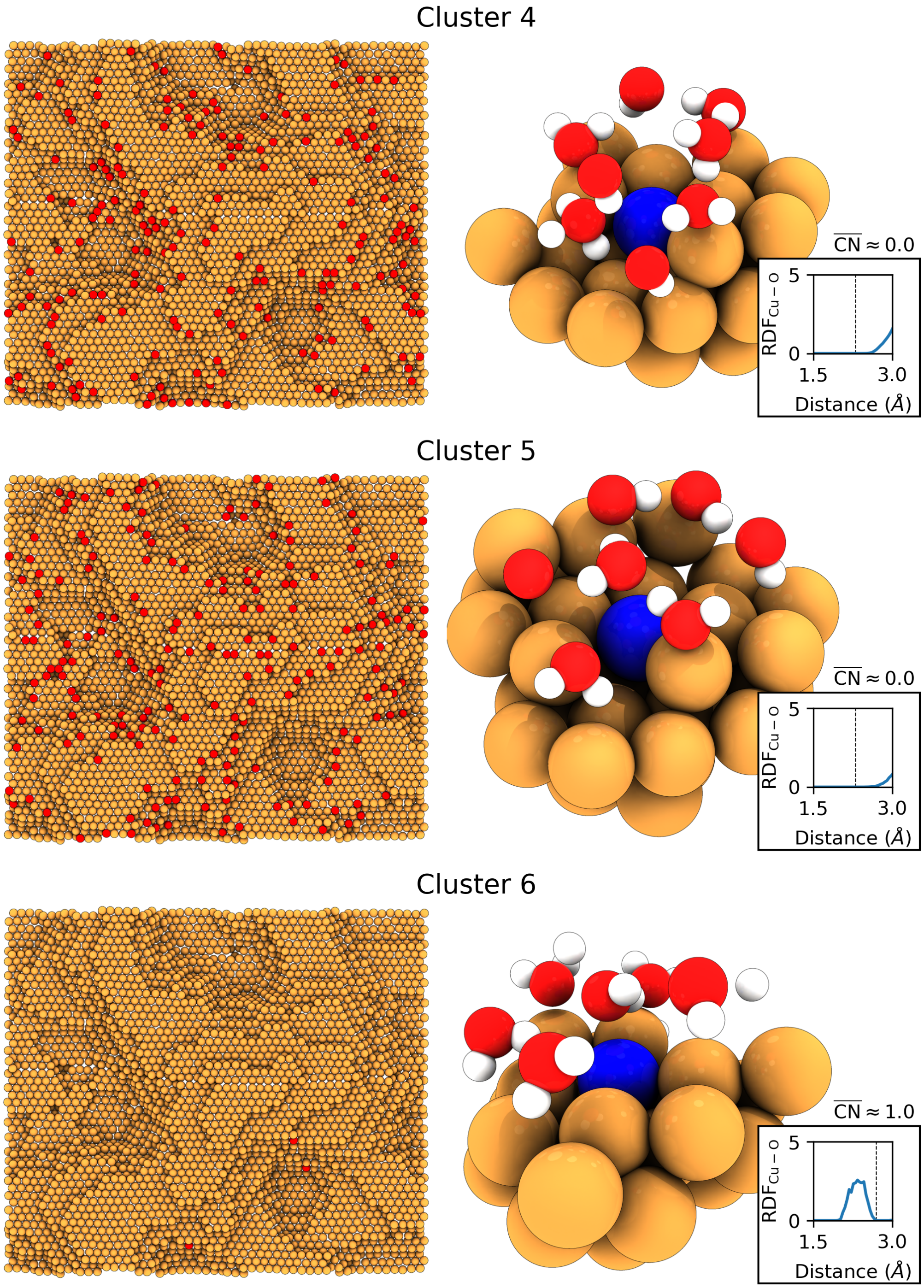

Figure S6: **Rough copper surface with atoms marked according to their cluster classification.** The left side of the figure depicts the rough copper surface, with atoms colored according to their cluster assignment as specified in Figure 6. On the right, an exemplary environment within a 5 Å radius of a cluster atom (marked in blue) is shown. In this representation, copper atoms are colored brown, oxygen atoms are colored red, and hydrogen atoms are colored white. Additionally, the average radial distribution function between copper atoms in this cluster and oxygen atoms is plotted. The average coordination number of copper with respect to oxygen is determined by integrating the first peak of the radial distribution function up to the point indicated by the black line.

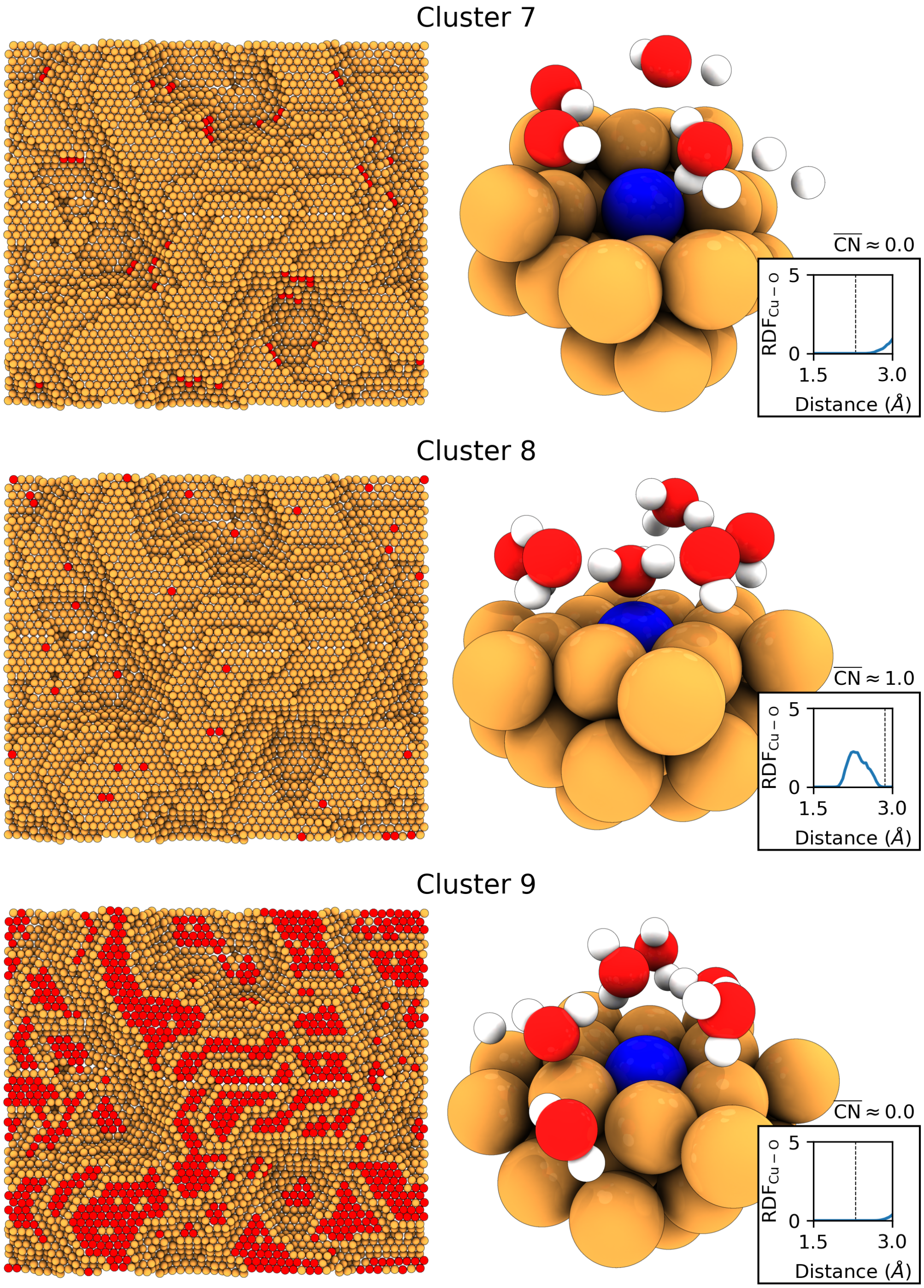

Figure S7: **Rough copper surface with atoms marked according to their cluster classification.** The left side of the figure depicts the rough copper surface, with atoms colored according to their cluster assignment as specified in Figure 6. On the right, an exemplary environment within a 5 Å radius of a cluster atom (marked in blue) is shown. In this representation, copper atoms are colored brown, oxygen atoms are colored red, and hydrogen atoms are colored white. Additionally, the average radial distribution function between copper atoms in this cluster and oxygen atoms is plotted. The average coordination number of copper with respect to oxygen is determined by integrating the first peak of the radial distribution function up to the point indicated by the black line.

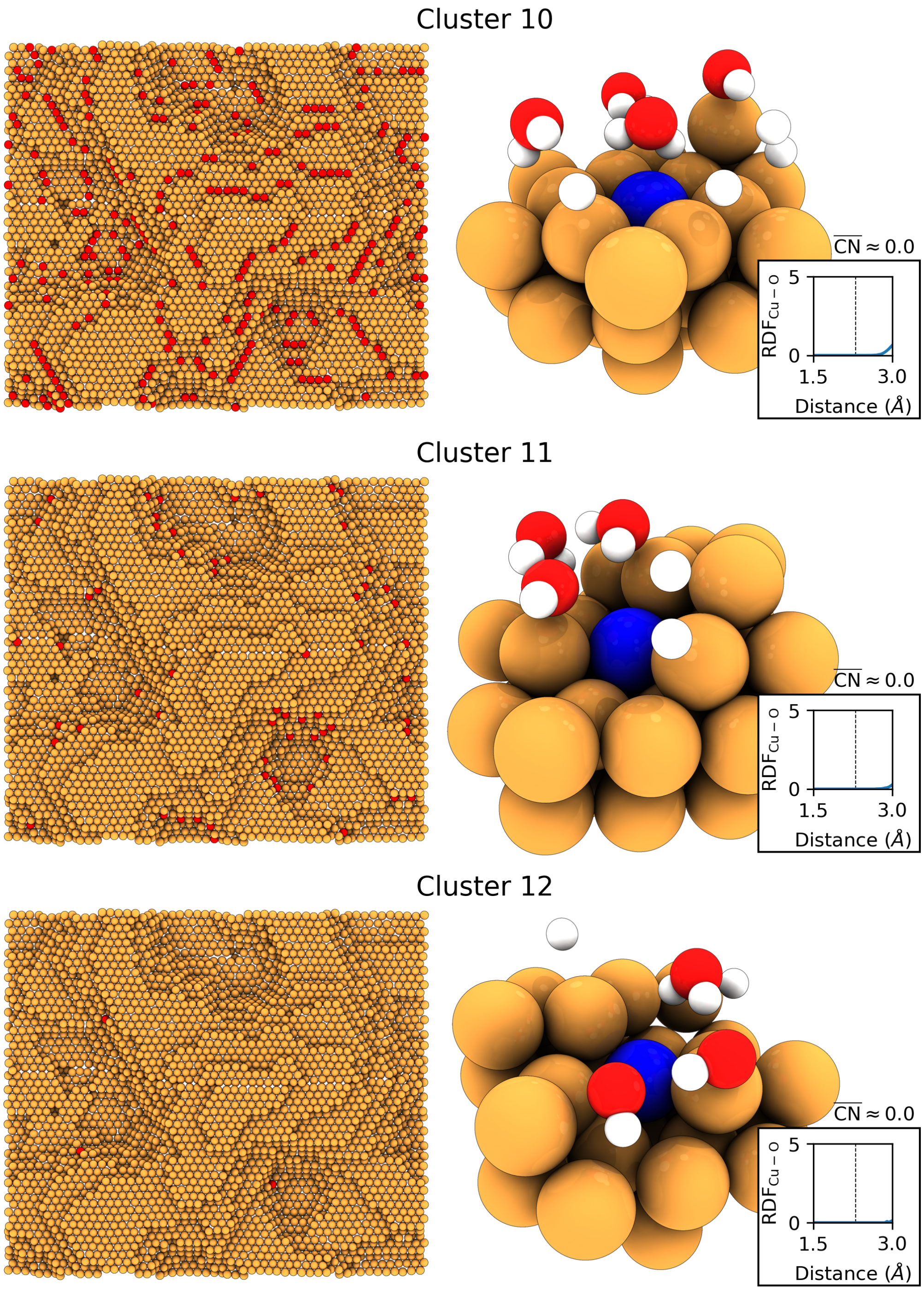

Figure S8: **Rough copper surface with atoms marked according to their cluster classification.** The left side of the figure depicts the rough copper surface, with atoms colored according to their cluster assignment as specified in Figure 6. On the right, an exemplary environment within a 5 Å radius of a cluster atom (marked in blue) is shown. In this representation, copper atoms are colored brown, oxygen atoms are colored red, and hydrogen atoms are colored white. Additionally, the average radial distribution function between copper atoms in this cluster and oxygen atoms is plotted. The average coordination number of copper with respect to oxygen is determined by integrating the first peak of the radial distribution function up to the point indicated by the black line.

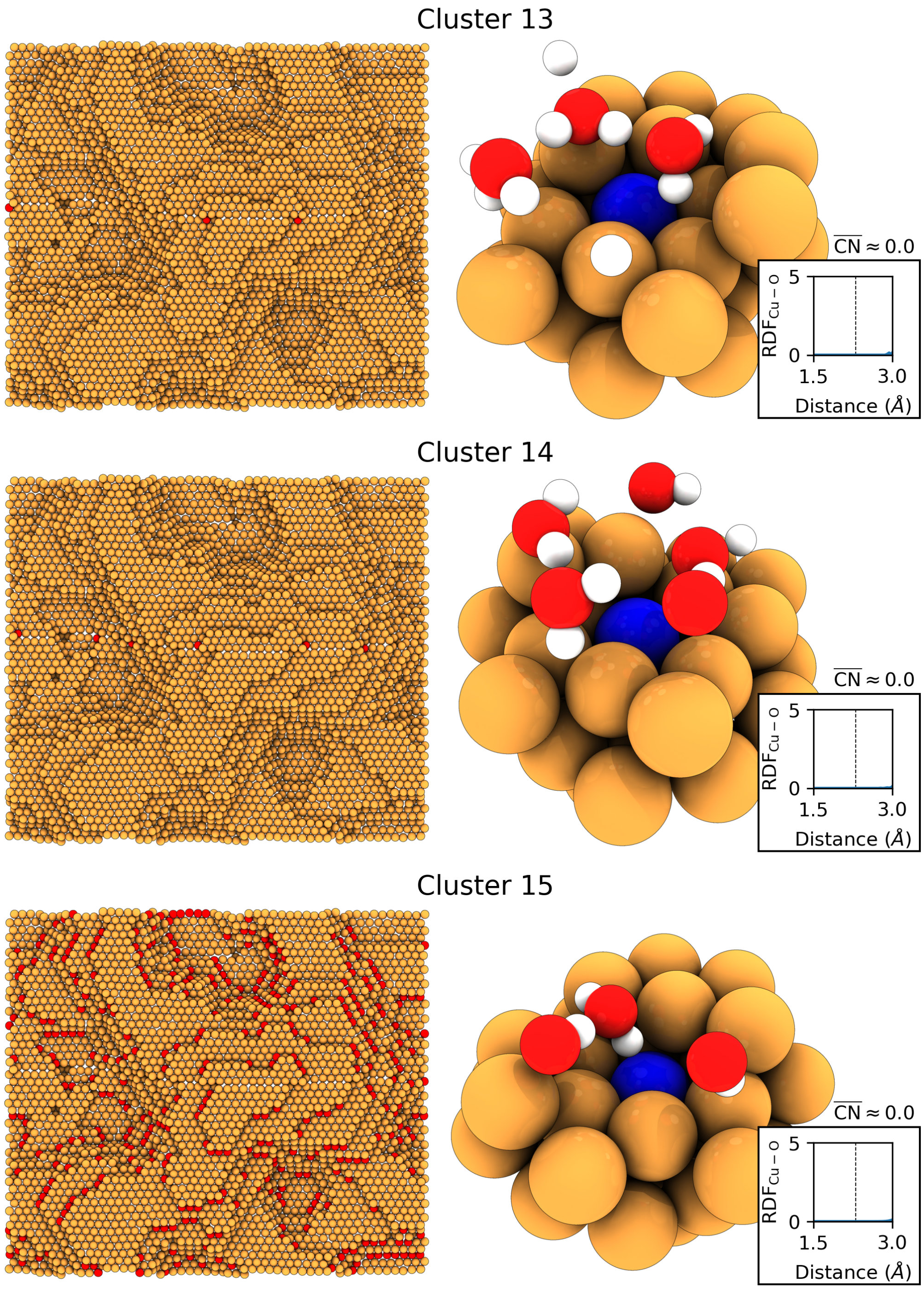

Figure S9: **Rough copper surface with atoms marked according to their cluster classification.** The left side of the figure depicts the rough copper surface, with atoms colored according to their cluster assignment as specified in Figure 6. On the right, an exemplary environment within a 5 Å radius of a cluster atom (marked in blue) is shown. In this representation, copper atoms are colored brown, oxygen atoms are colored red, and hydrogen atoms are colored white. Additionally, the average radial distribution function between copper atoms in this cluster and oxygen atoms is plotted. The average coordination number of copper with respect to oxygen is determined by integrating the first peak of the radial distribution function up to the point indicated by the black line.

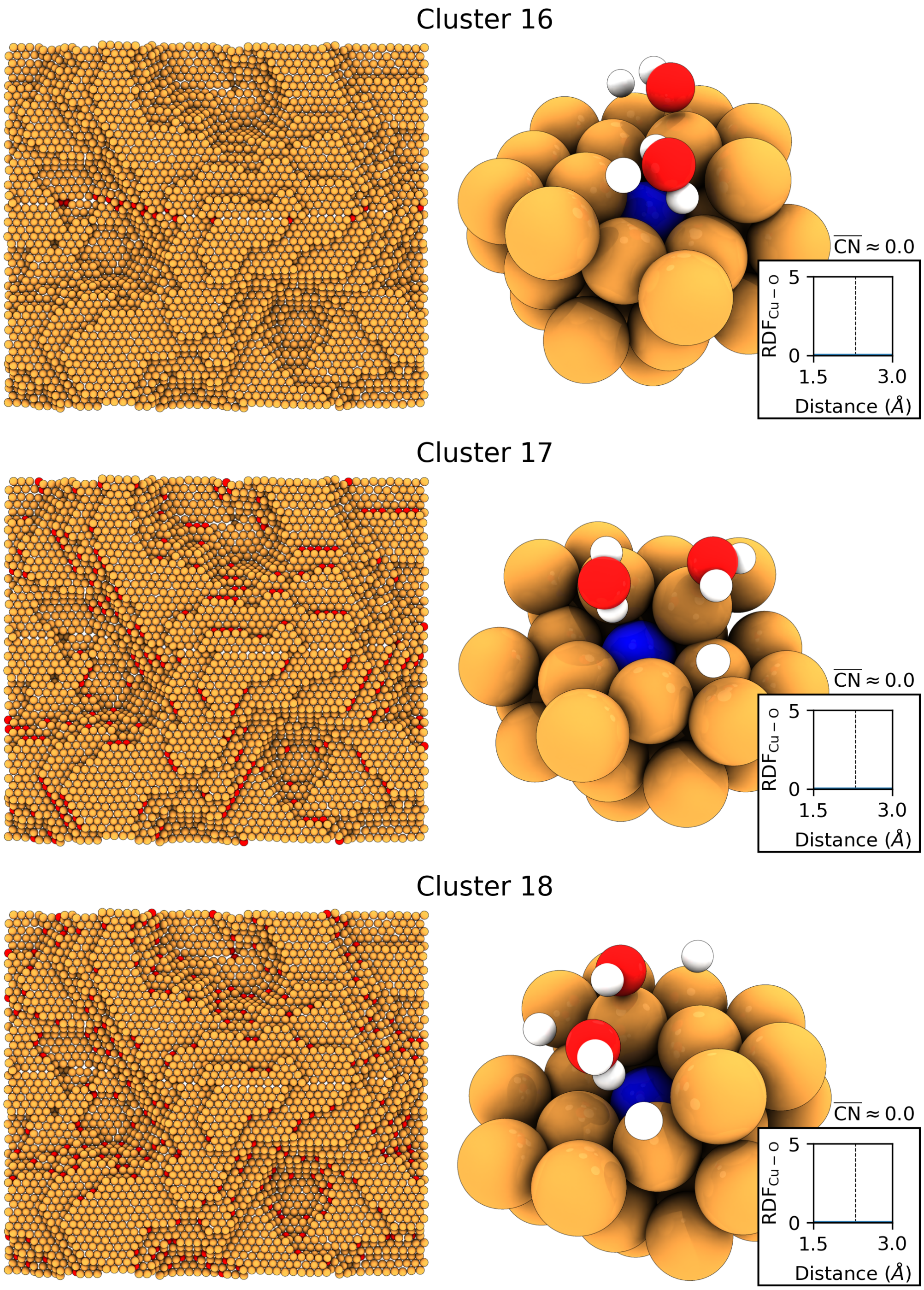

Figure S10: **Rough copper surface with atoms marked according to their cluster classification.** The left side of the figure depicts the rough copper surface, with atoms colored according to their cluster assignment as specified in Figure 6. On the right, an exemplary environment within a 5 Å radius of a cluster atom (marked in blue) is shown. In this representation, copper atoms are colored brown, oxygen atoms are colored red, and hydrogen atoms are colored white. Additionally, the average radial distribution function between copper atoms in this cluster and oxygen atoms is plotted. The average coordination number of copper with respect to oxygen is determined by integrating the first peak of the radial distribution function up to the point indicated by the black line.

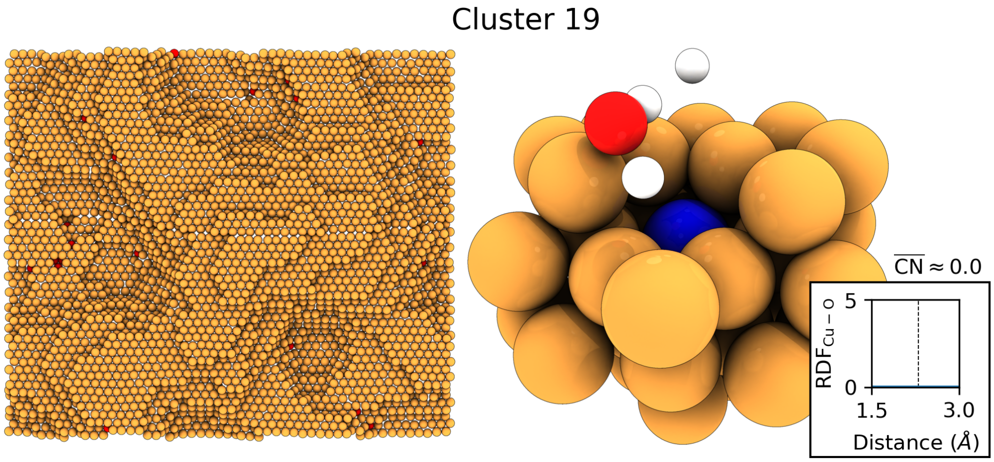

Figure S11: **Rough copper surface with atoms marked according to their cluster classification.** The left side of the figure depicts the rough copper surface, with atoms colored according to their cluster assignment as specified in Figure 6. On the right, an exemplary environment within a 5 Å radius of a cluster atom (marked in blue) is shown. In this representation, copper atoms are colored brown, oxygen atoms are colored red, and hydrogen atoms are colored white. Additionally, the average radial distribution function between copper atoms in this cluster and oxygen atoms is plotted. The average coordination number of copper with respect to oxygen is determined by integrating the first peak of the radial distribution function up to the point indicated by the black line.

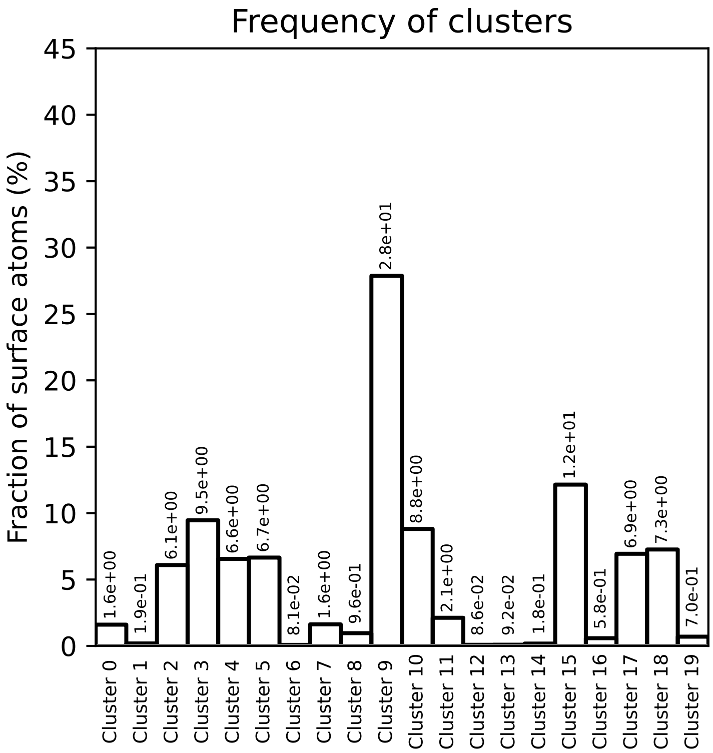

Figure S12: **Proportion of clusters on the rough copper surface.** Shows how many of the data points from Figure 6 are part of which cluster. Cluster 0 corresponds to noise data points, which have not been assigned to any cluster.

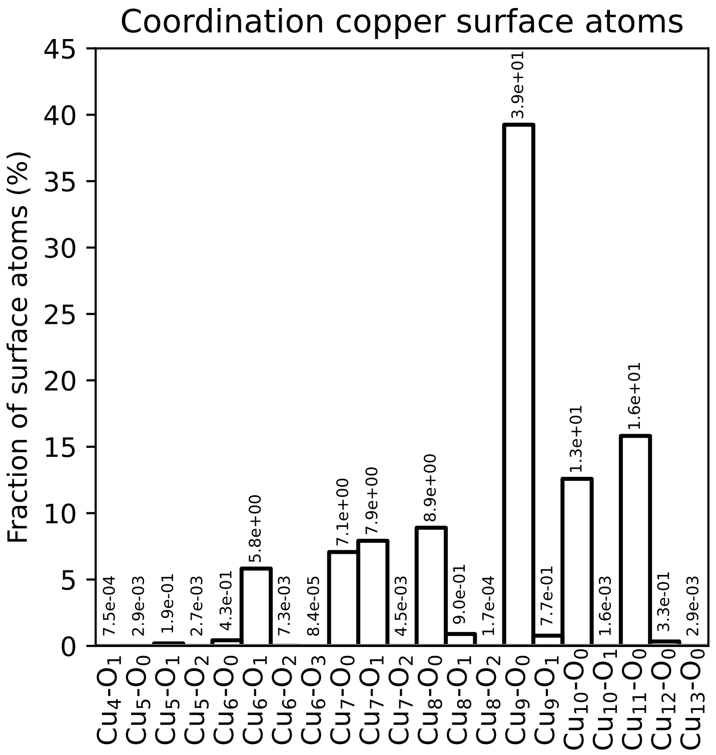

Figure S13: **Coordination numbers of the surface atoms.** Coordination numbers of all copper surface atoms to other copper and oxygen atoms. For copper-copper we used a cutoff radius of 3.25 Å, for copper-oxygen we used a cutoff radius of 2.5 Å.

Table S3: **Cluster classification.** Classification of all clusters from Figure 6 in the corresponding parts on the rough copper surface. For each cluster, we also note whether there is water chemisorbed.

Cluster	Position	Water chemisorbed
#1	Corner of a (111) plane with two neighbors in the same plane.	Yes.
#2	Corner of a (111) plane with three neighbors in the same plane.	Yes.
#3	Edge of a (111) plane with four neighbors in the same plane. Partially atoms at the stacking fault.	Yes.
#4	Edge of a (111) plane with four neighbors in the same plane.	No.
#5	Edge of a (111) plane with five neighbors (nearly completely surrounded by copper atoms).	No.
#6	(100) surface like facets.	Yes.
#7	(100) surface like facets.	No.
#8	(111) surface.	Yes.
#9	(111) surface.	No.
#10	(111) surface not directly below an edge, but close to.	No.
#11	(100) surface like facets directly below edges.	No.
#12	Rather complex edge/corner arrangements.	No.
#13	Directly below edges at the stacking fault.	No.
#14	Directly below edges at the stacking fault.	No.
#15	Directly below edges at (111) surface facets.	No.
#16	Closely below surface at stacking fault.	No.
#17	Directly below edge of a (111) surface facet.	No.
#18	Below (111) surface corners.	No.
#19	In small holes nearly below the surface.	No.
